# A Comparison of Peak Intensity Periods across Male Field Hockey Competitive Standards

**DOI:** 10.3390/sports9050058

**Published:** 2021-04-29

**Authors:** Eoin Cunniffe, Adam Grainger, Walter McConnell, Ulrik McCarthy Persson, Eamonn Delahunt, Colin Boreham, Catherine Blake

**Affiliations:** 1 School of Public Health, Physiotherapy and Sports Science, University College Dublin, Belfield, Dublin 4, Ireland; ulrik.mccarthypersson@ucd.ie (U.M.P.); eamonn.delahunt@ucd.ie (E.D.); colin.boreham@ucd.ie (C.B.); c.blake@ucd.ie (C.B.); 2 High-Performance Department, Hockey Ireland, Belfield, Dublin 4, Ireland; adam.grainger@hockey.ie; 3 Data Analytics, Backfour Analytics, Clontarf, Dublin 3, Ireland; wal@backfour.com

**Keywords:** external load, global positioning systems, match analysis, field hockey, team sports

## Abstract

This investigation aimed to compare the international level peak intensity period of male field hockey players to those experienced during professional and amateur club hockey match play. Twenty-seven players from an international squad were monitored for all activity relating to field hockey over three seasons. The peak intensity period, of 3 min duration, was extracted from match play files for international and club matches. Club matches were categorised by league standard—professional vs. amateur. The output for the peak intensity period, within positions, was compared using linear mixed models (LMMs) and post hoc pairwise comparisons. Significance levels were set as *p* < 0.05 and Cohen’s d was utilised for effect sizes. Competition level had a main effect on relative total distance *(p* < 0.05) and significant interaction effects were found between competition level and position (*p* < 0.05). Midfielders competing in amateur leagues and international match play completed less relative total distance than those who compete in professional leagues (−47.88 m/min, *p* < 0.05), (−46.06 m/min, *p* < 0.05) with large effect sizes reported. No other position displayed significant differences for peak periods. Match play in professional leagues provide opportunities for midfielders to experience peak intensity periods of a greater magnitude than international match play.

## 1. Introduction

Field hockey is a dynamic intermittent team sport that imposes unpredictable demands on players through a mix of aerobic and anaerobic efforts. This invasion sport is characterised by frequent short bursts of high-speed activities. When compared to soccer, rugby union and rugby league, field hockey players compete at a higher intensity, defined as relative total distance (RTD). However, the overall volume of physical output for metrics such as accelerations, total distance and high-speed running, during match play is lower for field hockey than other sports, due to match duration and rotational strategies [1,2]. As field hockey has unlimited substitutions, short periods of high-intensity activity occur frequently across a match, which interacts with the physical and technical output of the players [3]. However, the majority of field hockey research has focused on reporting full match averages from tournaments or splitting match data into halves or quarters [4].

While useful in providing reference values for the demands of match play and the associated variance, the role of rotation strategy, rotation length and shorter periods of intense play is omitted in these studies. Shorter epochs such as in-quarter rotations have been shown to impact physical and technical output, emphasising the importance of analysing shorter periods of play within quarters in field hockey.

In other sports such as rugby union and soccer, shorter epochs, known as the peak intensity period, have been investigated [5,6,7]. These periods of activity represent the peak demands of a match, for a selected metric, for a selected epoch e.g., most distance covered in a three-minute period. This is an attempt to model the peak running capacity of athletes during competition in order to better inform training practice [8].

During training, it is common to attempt to overload a specific metric i.e., relative total distance (m/min) through specific training drills, in order to prepare players for this peak period. Relative total distance is the most commonly utilised metric, due to its frequency of use in high level sport GPS-based research and training drill design [4,9,10,11].

These peak periods are relatively unexplored in field hockey research. However, insights gained from analysis of peak intensity periods, identified in this case as the most amount of distance covered in a defined period of time (3 min), can be used to inform conditioning and training practices and thus may provide a competitive advantage [12]. Furthermore, evidence that peak intensity periods are associated with key points of match play [13] further justifies the need for continued evaluation. Further support for this assessment process is demonstrated since it is known there is a reduction in the number of involvements, the quality of skill displayed, and a decrease in the distance covered, post the peak period of play in rugby league [14,15]. Meanwhile, it is worth noting that an increase in the frequency of rotations in field hockey increased the technical actions of forwards, through increased in-match recovery periods, by allowing greater physical output and interaction with the ball subsequent to a recovery period, which further supports the notion that fatigue may play in a role in field hockey technical performance [3]. This potentially identifies an optimal time to rotate players to prevent this decline in performance from occurring during match play.

At club level, in the Australian Hockey League, 1–10-min peak periods were investigated, with peak RTD emerging as 199 m/min for a one-minute period and 130 m/min for 10 min [16]. Given the Australian Hockey League is a mix of international standard players and non-international standard players, this result might underestimate the peak period RTD in international hockey since non-internationals have been shown to complete less high speed running than their international level counterparts [17] and have a lower VO_2_ [18]. Therefore, an investigation based exclusively on international level players, integrating their data while participating at club and international level, is required. Players who compete at international level in field hockey have unique scheduling and physical demands, as similar to international soccer, the club and international season overlap (Table 1). The aim of an international team’s physical conditioning staff is to ensure that players have the physical capacity to cope with the peak demands of international match play; however, published reference data to inform this approach are not available from international match play. Furthermore, the published data in the area relate to Australian hockey which has a different playing style to teams based in Europe. Therefore, the currently published reference data have limited utility to international team practitioners in Europe. Given that international players, from the same international team, can play club hockey in several different leagues, of different standards, around the world, it is important to establish the peak intensity period of match play at the three different levels that international players are exposed to (amateur, professional and international).

Consequently, this paper aimed to establish and compare the peak intensity period from local amateur level club hockey, professional-level club hockey and international level match play, defined by relative total distance, utilising a single cohort of international representative players.

## 2. Materials and Methods

A longitudinal observational study involving male field hockey players was carried out and reported according to STROBE guidelines [19]. Players were eligible for inclusion if they were elite international field hockey players representing a Senior Men’s national team while concurrently either playing in professional hockey leagues or the amateur league. All participants gave informed consent to participate in this study and the study conformed to the Declaration of Helsinki. Only players who participated in a minimum of five international matches and ten club level matches across a three-season cycle were included in this study. These inclusion criteria ensured that the players investigated were frequently part of the international squad and were exposed to the normal loading patterns of an international level hockey player.

The time period for data collection relates to 2017/18, 2018/19 and 2019/20 club seasons, with international data captured during the same time period. Each participant wore an individual GPS unit (STATSports APEX), operating at 10 Hz, as part of their normal monitoring as members of the international field hockey panel. The reliability and validity of these units have been reported previously [20].

Twenty-seven players (mean age = 26 ± 4, mean maximal aerobic speed = 4.85 ± 0.13 m/s, mean years in squad (6 ± 4)) met the inclusion criteria and were classified into three positions: defenders (*n* = 9), midfielders (*n* = 10) and forwards (*n* = 8). Players were placed in their positions relative to their international selected position and average position on the pitch—obtained from the GPS unit post-match. In the first season, 22 players played in the amateur league while 5 played in a professional league. In the second season, 10 players transferred to professional leagues. In the third season, 9 players played in the amateur league while 15 players played in professional leagues with 3 players from the cohort retiring. The professional league players were based in the top division of club field hockey in Germany, The Netherlands and Belgium. Thus, while the available sample for the study consisted of one international team, multiple data points were generated for analysis by each player over a 3-year period.

Match data corresponding to official fixtures released by the official hockey league representatives was analysed. All non-active time was removed from analysis and accuracy for each dataset was checked against official timesheets and video (when possible) for short corners, injuries and goals scored. While at club level the match clock is not stopped for short corners and goals, it is relevant to remove this to reflect the true demands of the sport. All matches took place on artificial turf field hockey pitches (91.4 m × 55.0 m). Club matches were 70 min in duration split into four quarters of 17:30 min while international matches were 60 min split into four quarters of 15:00 min.

All players were instructed to turn on the GPS units 20 min prior to the usage in order to achieve satellite locking, with a horizontal dilution of precision of 0.86 ± 0.29 and a high number of satellites present 22 ± 0.9, indicating good satellite geometry [21]. All players utilised the same unit throughout the study to reduce variability. Units were placed in a bespoke neoprene vest by the individual players. When the researcher was present, the application technique of the players was assessed to ensure the fidelity of data collection. This occurred on a weekly or biweekly basis depending on the stage of the season. The unit was placed in the mid-thoracic area between the scapulae. Data from each unit were downloaded directly post-match using the STATSports Apex Pro Series Software (STATSports, Newry, Co. Down, Northern Ireland).

The peak intensity period of all matches was calculated in the bespoke STATSports GPS Software (STATSports, Newry, Co. Down, Northern Ireland), using the moving averages method [7]. The moving averages method requires the analysis of the raw instantaneous data, which in this case is a 10 Hz device that provides 10 instantaneous speed points per second. To determine the peak demands using this method, a moving average of 3-min duration is taken from the raw data. For example, for 3-min periods a moving average of 1800 data points (180 s with ten samples per second) would be calculated from the start to the end of each rotation for which the player was on the pitch and active, i.e., 0–1800, 1–1801, 2–1802, 3–1803, etc., for the duration of the rotation, and the peak 3 min period identified from this [16,22]. The time period of 3 min was utilised due to the stop-start nature of field hockey. The frequency of short corners awarded, the use of rolling substitutions and video referrals at international level as well as unpublished data from the principal investigator using GPS data of player activity highlighted 3 min as the most frequent length of unbroken activity. Relative total distance was chosen to identify the period given that this is a widely utilised metric in the team’s analysis of performance and training prescription.

### Statistical Analysis

Since it is well established that physical demand varies by position, analysis focussed on differences in physical output between (1) Competition and (2) the Competition X Position interaction, which were assessed using linear mixed models (LMMs). LMMs was utilized to overcome the assumption of independence of the repeated measures within individual players, within seasons and also due to the flexibility that this method has in accounting for the altering sample sizes between groups [23].

Several iterative models were constructed to identify the optimal statistical model. Potential fixed and random effects (random intercept and slope) were added sequentially with iterations of the model tested for best fit. Likelihood ratio tests were completed on the iterative models utilising the car [24] ANOVA function in R [25] to identify if models were statistically different from one another. Marginal and conditional r^2^ were assessed [26] for each model with both Akaike’s information criterion (AIC) and r^2^ informing model choice. If the addition of a random effect did not improve AIC, it was removed from the analysis process [27].

The dependant variable was physical output i.e., relative total distance. In all models, random effects included repeated measures of the player within seasons and within competitions. Random intercepts for participant, competition and season were thus generated to allow for the uniqueness of individuals, and the characteristics of each competition and season. Attempts to model random slopes for variables resulted in overfitting of models and was therefore discarded from the analysis.

The key variables of interest, Position (Defender, Midfielder, Forward) and Competition Level (amateur, Professional and International) and their interactions were included in the final model. All models estimated parameters using the restricted maximum likelihood method [28].

The LMM’s were computed in R, using the package lme4 [29]. Model performance was tested utilising the ‘performance’ package [30]. Statistical significance was accepted where *p* < 0.05. Post hoc pairwise comparisons between different positions or competition level were carried out where appropriate, using Bonferroni adjustment to the threshold for statistical significance with the emmeans package [31]. Mean differences and the respective standard error (SE) of measurement were reported between groups. Effect sizes (ES) for significant differences were also determined using Cohen’s d statistic. Effect size values of ≥0.20, ≥0.60, ≥1.20 and >2 were considered to represent small, moderate, large and very large differences, respectively [32].

## 3. Results

Four hundred and forty individual peak intensity periods were analysed for the 27 players, with an average of 16 ± 10 periods per individual. For international matches, amateur club matches, and professional club matches 191, 132 and 117 peak periods were analysed, respectively. The number of samples analysed for midfielders, forwards and defenders were 205, 146 and 89 respectively. Estimated marginal means for each position and competition can be found in Table 2.

### 3.1. Competition Main Effect

A significant main effect (fixed effect in model) was found for competition level for the RTD metric (*p* < 0.05). Amateur club level displayed significantly lower RTD completed compared to the professional leagues (mean −33.98 m/min, SE = 7.44, *p* < 0.05, ES = 0.60). Similarly, international level output displayed significantly lower RTD in comparison to the professional leagues (mean −29.51 m/min, SE = 6.98, *p* < 0.05, ES = 0.62).

### 3.2. Interaction between Competition and Position

**Figure 1:**
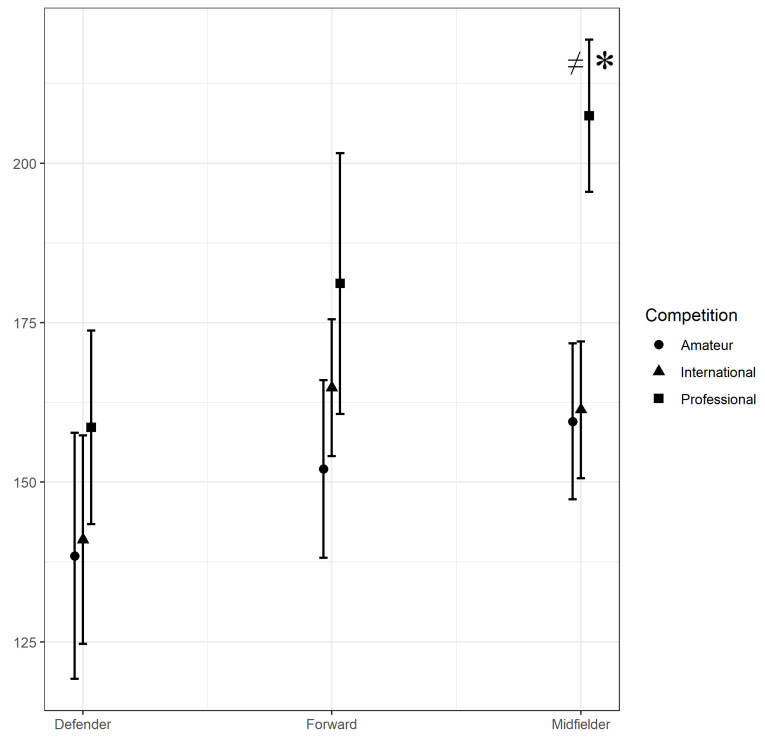
Estimated marginal means for RTD by Position and Competition with annotation for statistically significant differences. * = statistically significantly different to International and ≠ = statistically significantly different to Amateur.

Significant interaction effects were found between Competition * Position for RTD (*p* < 0.05). Midfielders competing in amateur leagues completed less RTD (mean −47.88 m/min, SE = 8.58, *p* < 0.05, ES = 1.31) (Figure 1) than Midfielders who competed in professional leagues. Midfielders during international matches, also completed less RTD than those competing in professional league match play (mean −46.06 m/min, SE = 7.43, *p* < 0.05, ES = 1.25) (Figure 1). While a similar pattern was found between competition level RTD for both Defenders and Forwards, neither was statistically significant.

## 4. Discussion

This is the first study to compare the peak intensity periods across competitive levels in field hockey. The aim was to determine if the demands vary between different playing standards, identifying if professional or amateur levels replicate or exceed that of international match play regularly. The relative total distance values identified in this investigation may provide practitioners with guidelines for training drill prescription that closely replicate match play at several competitive levels for a specific time period in field hockey.

Considering competition level irrespective of position (main effect), professional league competition elicited the greatest output for RTD, an arbitrary measure of match intensity, with both international and amateur level displaying significantly less RTD. This was unexpected, as it was assumed that International level would surpass both levels of club match play given that there is ten minutes less total match play in international hockey matches which may allow for a reduction in the need for pacing [33]. Furthermore, players tend to complete longer duration rotations during club level match play. This, combined with the finding that club level players exhibit lower fitness levels than their international counterparts, may induce a reduction in physical output therein eliciting less intense peak periods, however, this was not the case [17]. While the Irish Hockey League is classified as ‘amateur’, it does, contain players who compete at the highest level of performance i.e., international level, which may increase the standard of the league. Interestingly, there were limited differences between amateur and international level which may point to international players being able to maintain their intensity when playing at amateur club level. However, the results of this investigation suggest that professional league surpasses both international and amateur level and therefore may be the optimal setting to ensure players both meet and exceed international level demands on a regular basis. This is contrary to what was anticipated. This might be explained by the fact that the professional league in this study refers to the Belgian, Dutch and German leagues which are the strongest leagues in the world. The majority of these teams are comprised of players who play for international teams who are currently ranked in the top five positions in the world [34]. In international matches, highly ranked teams have been shown to elicit greater physical output from the opposition [35]. This effect may be carried into the professional leagues through the predominance of players from highly ranked teams. Additionally, some of the international match data are from match play versus teams ranked >15th in the world which may lower the output values [34].

When the interaction between player position and competition is considered, the differences between competition levels are less clear, with only the position of midfielder showing statistical significance. Midfielders in the amateur league generate lower peak RTD values than midfielders participating in professional leagues. It is speculated that playing in a professional league may offer advantages for international midfielders, permitting them to experience intensities even greater than international level to ensure they are prepared for performance at international level. These findings are not present for other positions. Further exploration of how these peak values are achieved is required to determine the cause of the discrepancy, with the possibility existing that these periods may be accumulated through different physical output strategies i.e., midfielders in professional leagues may accumulate more high speed running within these periods [17].

Defenders, midfielders and forwards in amateur leagues and at international level display less relative total distance than their position matched counterparts in the Delves et al. [16] study which used participants from the top league in Australia. However, in professional leagues, only defenders displayed less (−3 m/min) whereas forwards and midfielders exceed the RTD completed (+15, +40 m/min) [16]. This may arise as the league, investigated by Delves et al. [16] is utilised as a national team selection competition, consisting mostly of prospective international level players. This international team is a higher-ranked team than the international team providing the data for this current study (+11 Ranking Positions). Potentially, professional leagues, in this investigation, exceed these levels as the players present are of the same standard but tend to play a different tactical style of hockey.

Typical values for relative total distance in international field hockey match play have previously been reported as 120–141 m/min for a full match [36,37]. Given that international peak periods found were, on average, 157 m/min for a three-minute period, using total match average data to inform training practices may underrepresent the true scale of match demands and inadequately prepare players for important phases of play. This may be particularly the case when position is considered; for example, for forwards and midfielders’ reliance on average match average data, could misrepresent the true intensity demanded by up to 20%.

This investigation is not without limitations. The international data utilised are from a single team and their unique style of play is largely influential on the values reported. A team with a different style of play may produce peak intensity periods that are contrasting to those reported. Additionally, the data utilised to represent club level match play originated from individuals who competed for a broad group of clubs within those leagues who have a varied style of play. However, this could be viewed as a strength as it provides a wide representation of clubs within the leagues investigated and makes the findings for that setting applicable to a wider array of practitioners. Furthermore, only the physical output of players was reviewed in this investigation with no consideration of the tactical and technical context in which this physical output was completed. The authors acknowledge that performance is a much wider construct than physical output alone, however, the authors believe establishing these values is an important foundational step for further contextualised field hockey research relating to the peak intensity period.

The current investigation is relevant as peak periods have been shown to be match-defining moments [13] and are often utilised to set parameters and targets for drills in training to replicate the true intensity of match play [38]. This approach was trialled in field hockey with small-sided games, both meeting and exceeding the peak acceleration demands of match play. However, small-sided games failed to match the demands with respect to RTD [10]. Similarly, in soccer, Martin-Garcia et al. [38] showed small-sided games could match and exceed peak match play acceleration demands but not match the peak total distance, high metabolic load distance, average metabolic power, high speed running or sprint parameters reached in match play. This may underline that match play provides unique constraints and scenarios that are difficult to recreate in a training environment. Therefore, player placement in the competitive environment i.e., professional versus amateur leagues should be considered as a strategy to ensure they are exposed to peak physical demands on a frequent basis.

An applied example of how these data have been used in an international training environment is each drill used in the training environment, of the same duration using relative total distance as a reference metric, has been analysed and converted to a percentage of the peak values achieved at each competitive level. This allows for an analysis of whether players require further conditioning to ensure the threshold is met during training. A common question asked by coaches is “did we meet match intensity in that training session?” and these values provide reference points to answer this question. Similarly, they act as a feed-forward training design process as international team practitioners are now aware of the expected values that players will have experienced at club level which allows for practitioners to narrow their focus to different elements of performance. I.e., midfielders in professional leagues do not require a focus on reaching these targets in international training scenarios as they are already experiencing them. A further practical use of this data for practitioners of international teams would be to prescribe drills, for midfielders who participate in amateur leagues, that produce peak intensity period values similar to that of professional and international match play to prepare players to meet the demands of international match play. These drills may need to be large sided games due to the difficulties reported in reaching these targets with small sided games [38].

Regarding future research based on the findings of this investigation, it would be worthwhile to investigate the experiences of players who provided the data for this study and their perception of these periods at each level. A large proportion of the participants competed in both the amateur and professional leagues during the investigated period. Ascertaining whether the players perceived the differences identified and whether midfielders in the professional leagues felt more prepared for the peak intensity periods of international match play would add ecological validity to this investigation. Additionally, greater context is required relating to these periods and how they are accumulated. Future research should focus on establishing the tactical and technical components of match play that leads to the occurrence of these periods. Finally, the impact of these periods on performance, in terms of how they influence individual player performance should be examined.

## 5. Conclusions

In conclusion, the main finding was that interaction effects between position and competition indicate that midfielders are mostly affected by competition level, with a greater peak RTD experienced in professional leagues compared to both amateur and international level. No other position displayed any significant difference between peak RTD achieved. The practical relevance of this finding relates to training scenarios where players competing at professional level may be better prepared to deal with the demands of peak intensity periods that occur during international match play. Additionally, the values reported for all levels provide benchmarks for each level of competition that practitioners may use to prescribe and monitor training to ensure training provides exposure similar to that of match play.

## Figures and Tables

**Table 1:** Example of a Two-Week Schedule of a Professional Hockey Player in season 6 weeks before World Cup 2018.

Type	Day
Club Match	1
Day Off	2
Club Match & Travel	3
International Match	4
International Match	5
Gym	6
International Match	7
International Match	8
Day Off	9
International Match & Travel	10
Day Off	11
Club Training & Gym	12
Recovery Day	13
Club Match	14

**Table 2:** Estimated marginal means (SE) for the peak intensity periods by competition and position.

Competition	Position	RTD (m/min)
Professional	Defender	158.59 (7.81)
International	Defender	141.01 (8.38)
Amateur	Defender	138.48 (9.89)
Professional	Midfielder	207.41 (6.17)
International	Midfielder	161.35 (5.54)
Amateur	Midfielder	159.53 (6.31)
Professional	Forward	181.14 (10.57)
International	Forward	164.82 (5.54)
Amateur	Forward	152.08 (7.13)

## Data Availability

The data presented in this study are available on request from the corresponding author. The data are not publicly available due to the sensitivity of the data relating to the performance of an international field hockey team and within organization data protection and policy standards.
